# On the Coupling between Mechanical Properties and Electrostatics in Biological Membranes

**DOI:** 10.3390/membranes11070478

**Published:** 2021-06-28

**Authors:** Vanesa Viviana Galassi, Natalia Wilke

**Affiliations:** 1 Facultad de Ciencias Exactas y Naturales, Universidad Nacional de Cuyo, Mendoza M5500, Argentina; vgalassi@mendoza-conicet.gob.ar; 2 Instituto Interdisciplinario de Ciencias Básicas (ICB), Universidad Nacional de Cuyo, CONICET, Mendoza M5500, Argentina; 3 Departamento de Química Biológica Ranwel Caputto, Facultad de Ciencias Químicas, Universidad Nacional de Córdoba, Córdoba X5000HUA, Argentina; 4 Centro de Investigaciones en Química Biológica de Córdoba (CIQUIBIC), Universidad Nacional de Córdoba, CONICET, Córdoba X5000HUA, Argentina

**Keywords:** lipid ionization, electric field, flexoelectricity, electro-mechanical properties, electroporation, nerve impulse

## Abstract

Cell membrane structure is proposed as a lipid matrix with embedded proteins, and thus, their emerging mechanical and electrostatic properties are commanded by lipid behavior and their interconnection with the included and absorbed proteins, cytoskeleton, extracellular matrix and ionic media. Structures formed by lipids are soft, dynamic and viscoelastic, and their properties depend on the lipid composition and on the general conditions, such as temperature, pH, ionic strength and electrostatic potentials. The dielectric constant of the apolar region of the lipid bilayer contrasts with that of the polar region, which also differs from the aqueous milieu, and these changes happen in the nanometer scale. Besides, an important percentage of the lipids are anionic, and the rest are dipoles or higher multipoles, and the polar regions are highly hydrated, with these water molecules forming an active part of the membrane. Therefore, electric fields (both, internal and external) affects membrane thickness, density, tension and curvature, and conversely, mechanical deformations modify membrane electrostatics. As a consequence, interfacial electrostatics appears as a highly important parameter, affecting the membrane properties in general and mechanical features in particular. In this review we focus on the electromechanical behavior of lipid and cell membranes, the physicochemical origin and the biological implications, with emphasis in signal propagation in nerve cells.



**Contents**

**1      Introduction**

**2**

**2      Surface Charge Density, Surface and Zeta Potentials**

**4**

**3      pH Effects**

**5**

**4      Effects of Ions Different from Protons**

**6**
      4.1      Metal Cations6      4.2      Cationic Peptides9
**5      Potentials across Membranes**

**11**
      5.1      Electromechanical Coupling11      5.2      External Electric Fields and Electroporation13      5.3      Nerve Impulse Propagation15
**6      Summary and Future Perspectives**

**17**

**References**

**18**



## 1. Introduction

Cell membranes are self-assembled structures formed by lipids and proteins, and constitute a complex and constantly changing environment. The strength of the inter-molecular interactions defines the mechanical properties of these quasi-bidimentional systems, and these interactions may be Van der Waals forces (Keesom, Debye and London interactions [1]), hydrogen-bonding, and/or forces between permanent charges, dipoles or higher multipoles. It is clear that these last forces are electrostatic, but in truth all other forces of interaction also have an electrostatic origin, inflicted in essence by the charged nature of fundamental particles. Thus, electrostatics commands membrane mechanical properties.

Electrostatic forces among *N* particles are led by a simple equation, Coulomb law:

(1) $$\vec{F}=\sum_{i=1}^{N}k\frac{Q_{1}Q_{2}}{r^{2}}\hat{r}$$

Since electrostatic forces are additive, all electrostatic interactions in a system can be accounted for using this equation if the position of all charges are known. This apparently rather simple task involves the summation (or integration in case of charge densities) of all charges at each position which may turn out extremely complicated, depending on the spacial distribution of the charges.

An important parameter in Equation (1), which is included in *k*, is the local dielectric constant in the proximity of each charge. The dielectric constant ε is the ratio of the dielectric permittivity of the material to that of vacuum, and accounts for the degree of screening of an electric field by a material. In the interior of a material, ε may either be constant or vary from a region to the other. In the case of lipid bilayers, the dielectric constant varies from a value close to 80 (in the internal and external aqueous milieu) to a value close to 2 (in the bilayer interior). In the region of the polar headgroups, an intermediate value is expected that depends on the lipid class and phase state. In lipid monolayers, values in the range of 5–20 have been determined [2].

Cell membranes contain lipids and proteins, and thus different values of ε are expected along the membrane plane. In the regions of the membrane enriched with lipids, values close to 2 are expected in the middle of the bilayer and values close to that determined in lipid monolayers/bilayers for the region of the polar head groups. However, the dielectric constant of cytoplasm may be very different from 80 since water is not free to move but ordered inside cells due to molecular crowding, as has long been noted by different authors [3,4]. Furthermore, water order varies during cell metabolism [5] and, as all properties in cell interior, it is very likely to vary from a region to the other inside cells. Nonetheless, constant values of 80 [6,7,8] or 60 [9,10] are typically accepted as approximations.

Experimental determinations of ε in the cell interior have been performed in yeast, yielding values in the range 50–60 [11,12,13]. Values of ~60 have been found for erythrocytes [14], and ~70 for T-lymphocytes [15] and for viral tegument [16]. Very intriguing, values as high as ~90 have been reported for the dielectric constant of the cell interior in MEL cells, indicating the presence of an unidentified dielectric polarization mechanism capable of increasing the effective interior permittivity [17]. Authors suggested that the combined effects of several organelles may be responsible of the reported large value.

**Figure 1:**
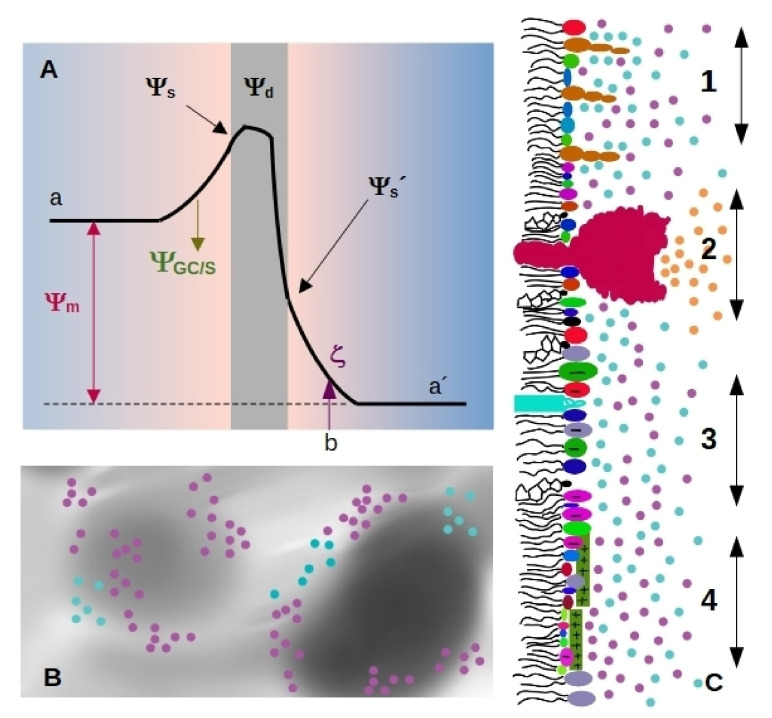
Scheme illustrating membrane electrostatics. (**A**) Electrostatic potential profiles that bear a membrane (gray region) along the normal axis. Volta potentials are usually different on each side of the membrane, the difference between the value on the left (a) and right (a’) side corresponds to the membrane potential $\Psi_{m}$. Charged species on the membrane generate the surface potential $\Psi_{s}$. The charged membrane induce an ion cloud and a potential drop characterized through Gouy-Chapman or Stern model $\Psi_{GC/S}$. In the scheme, the slipping plane is at a distance b from the membrane, and the potential value at this point corresponds to the zeta potential ζ. Membranes are composed of multipoles organized in an ordered array, generating the dipole potential $\Psi_{d}$. (**B**) Possible charge distribution in the membrane plane with non-homogeneous electrical properties. Gray levels indicate different values for the dielectric constant. Cyan circles represent cations and pink circles, anions. (**C**) Lateral view of a membrane hemilayer, where different situations coexist. As in B, cyan circles represent cations and pink circles, anions. 1—Gangliosides protrude from the plane formed by the polar head groups of less bulky lipids, generating a rough surface. 2—A pump or a channel generates a local ion gradient (orange circles correspond to protons, sodium, calcium or the specific ion that passes trough the pump/channel). 3—Region of the membrane enriched in anionic lipids. This region will attract cations. 4—cationic peptides adsorbed to a region of the membrane generate a positive surface that attracts anions.

Charge densities generate electrostatic potentials with magnitudes that depend on ε. Four important electrostatic potentials can be described in biological membranes, which are schematized in Figure 1A [18,19]:

1. Membranes separate the interior from the exterior media, having different Volta potential. The difference between these values is called membrane, transmembrane or diffusion potential $\Psi_{m}$ [18], see Figure 1A (we will use the term membrane potential for $\Psi_{m}$ throughout the review). The absolute value of this potential difference is about $10^{1}$–$10^{2}$ mV and varies in distances of tens of nm, thus generating high electric fields (of the order of $10^{6}$–$10^{7}$ V/m) through the bilayer.
2. The presence of highly ordered molecules with a charge distribution characterized by a dipole or higher order multipoles inside the membrane, as well as a lower dielectric constant in the membrane interior compared to the surface, gives rise to a non-zero potential inside the membrane called dipole potential $\Psi_{d}$ [19]. This potential is positive in the center of the bilayer (Figure 1A), and leads to very high electric field inside the bilayer (of the order of $10^{-1}$V$/10^{-9}$m = 10${}^{8}$ V/m).
3. Charged moieties on the membrane surface generate a potential difference between the membrane surface and the solution called surface potential $\Psi_{s}$ (see Figure 1A). These charges in turn interact with small ions or charged molecules from the solution, resulting in an ion cloud around the membrane. The membrane surface charge, together with the ionic composition of the solution modulates the potential drop due to the ion cloud, and it can be modelled using Gouy-Chapman approach ($\Psi_{GC}$), or with Stern model ($\Psi_{S}$) in the presence of specific ion-membrane interactions [18], see Figure 1A. Gouy-Chapman model predicts an exponential decay on the potential as we move away from the charged surface, with a characteristic distance $\lambda_{D}$ (Debye lenght). Stern model predicts a linear drop in the region of adsorbed ions, and a behavior according to Gouy-Chapman model at larger distances. Both models are broadly used in membrane biophysics, working remarkably well despite having coarse approximations. With respecto to this, a good correspondence was found between ion distribution close to a phosphatidylglycerol membrane predicted by Gouy-Chapman model and obtained using molecular dynamics simulations [20].

The diffuse layer contains small anions and cations, including protons. In turn, pH defines ionization of acid and basic polar head-groups and thereby the membrane charge density. Therefore, there is a strong interdependence between membrane ionization and composition of the aqueous milieu close to the membrane [21,22,23].

Since membrane composition is not constant, dielectric constant and charge density are expected to vary from a region to the other, as schematized in Figure 1B. Therefore, each region of the membrane may bear different values of $\Psi_{s}$ and $\Psi_{d}$ (Figure 1C), which affect local interactions and thus, diffusional properties of the species within the membrane, local compressibility and bending, and local interaction between membrane and soluble charged species. Several membrane properties may be affected by pH or ionic composition. Among them, we can state melting temperature and phase state, local curvature, stiffness, and bending. These properties may change simultaneously or not, in a coupled or uncoupled manner, and changes are either subtle or marked. Therefore, how emergent properties in membranes behave upon environmental changes is an open question.

## 2. Surface Charge Density, Surface and Zeta Potentials

Depending on the particular membrane, 10 to 40% of the lipids are negatively charged. Cationic lipids such as sphingosine and psychosine are uncommon and are present in healthy cells in a very low proportion. Proteins inserted on the membrane or adsorbed to it may also bear charges. Both, anionic and cationic species lead to non-zero surface charge density, and to the presence of an ion cloud close to the surface whose density varies within the characteristic distance $\lambda_{D}$ (close to one nanometer at physiological conditions).

The surface charge adds an electrostatic term on the membrane free energy that depends on the transversal area of the charged species and on ionic strength. In general, the increase in surface charge density tends to expand the molecular area [23]. Since lipid area is related to membrane phase state, the temperature for phase transitions depends on the membrane charge density, and therefore is affected by the proportion of charged lipids in the membrane as well as by ionic strength [23,24]. Given that each phase state is characterized by its particular mechanical properties, it is expected that electrostatics influences the viscoelastic response of membranes upon different stresses. Aside from changes related to phase transitions, many other effects have been reported as detailed in the following sections.

Surface charge density can be studied by means of electrophoretic mobility, which gives information about electrostatics at the hydrodynamic plane of shear (slipping plane, SP). This plane is located at a distance $d_{SP}$ from the membrane surface, marked as *b* in Figure 1A). The potential at this point is called zeta-potential (ζ), and depends on $\Psi_{s}$, the drop in electrostatic potential due to adsorbed ions (Stern model), and screening by the ion cloud (Gouy-Chapman model).

If $d_{SP}$ is known, the Gouy-Chapman/Stern model can be used to estimate the value of the surface potential from zeta-potential measurements. However, $d_{SP}$ is not known with precision even though some estimations have been reported. A distance of 0.2 nm from the membrane surface was postulated early in 1979 by the group of McLaughlin for phosphatidylserine or phosphatidilglycerol vesicles in solutions containing alkali metal cations [25]. Later, in 1992, Egorova et al. proposed a similar value using a different experimental approach [26]. In another set of experiments, McLaughlin et al. claimed that $d_{SP}$ changes with ionic strength, going from 0.2 nm for 0.1 M to 1 nm for 0.001 M NaCl [27]. A value close to the nm was proposed by Simon et al. for phosphatidilcholine/cholesterol vesicles in 0.01 M KCl [28].

Considering that the diameter of a water molecule is about 0.2–0.3 nm [29,30], and that lipid membranes are not atomically flat, a value for $d_{SP}$ lower than the nm appears very small. Furthermore, lipids with a bulky polar headgroup (such as GM1) mixed with small charged headgroups will generate a surface roughness larger than the distance attributed to SP position as schematized in Figure 1C [27]. In this regard, Woodle et al. found that incorporation of pegylated phosphatidylserine (polyethylene glycol attached to the polar headgroup of the lipid) into bilayers produces a substantial negative surface potential, but an electrophoretic mobility lower than expected. This was explained considering an increase in the hydrodynamic radius due to steric effects of polyethylene glycol moieties [31].

Despite the uncertainty of $d_{SP}$ value, changes in ζ are sometimes taken as estimations of changes in $\Psi_{s}$. For instance, the affinity of cationic peptides to anionic membranes can be obtained from ζ determinations [32] provided $d_{SP}$ does not change and peptide adsorption does not cause ion displacement from the membrane surface. In this context, it is important to bear in mind that at physiological conditions $\lambda_{D}$ is in the order of one nm ($\lambda_{D}$ = 0.8 nm for 150 mM of NaCl for instance), which means that at these distances, the potential value will be 1/e = 0.37 times the value at the surface. Therefore, ζ determinations will yield lower values than $\Psi_{s}$ (see Figure 1A). Besides, it has been shown that ζ does not vary linearly with ionic strength [26], meaning that variations of ζ do not always change proportionally to changes in $\Psi_{s}$. Considering Gouy-Chapman model, the values of ζ and $\Psi_{s}$ will be more similar the higher is $\lambda_{D}$ than $d_{SP}$. For instance, $\zeta =0.9\Psi_{s}$ for conditions at which $\lambda_{D}$ is 10-times higher than $d_{SP}$. Therefore, ζ will better reflect surface potential in solutions of low ionic strength, which are unfortunately not physiologically relevant conditions.

## 3. pH Effects

The pH of the aqueous milieu regulates charge density by modifying the charge state of ionizable groups in the membrane. In this regard, the pKa range of different lipid classes are known [26,33,34,35,36,37] but chain length and unsaturation, as well as lipid clustering, affect the exact pKa value [23,38,39,40,41]. The presence of ions that interact specifically with the charged species (such as calcium and sodium) also affects the actual pKa value of the lipid in a membrane [42,43,44]. Besides, membrane composition may alter the pKa of polar headgroups by means of lateral interactions. In this regard, H-bonds between lipids and lipids with proteins have been shown to affect the pKa value of phosphatidic acid and of phosphoinositides [45,46,47]. Furthermore, charge saturation occurs in liposomes with high percentage of anionic lipids. A linear increase in charge and surface potential was reported only up to 20–30 mol% of charged lipids at physiological conditions, and up to lower proportions at low salt conditions [48]. With respect to protein charge, similar to what happens with lipids, the pKa of ionizable amino acids is highly dependent on the environment [49,50]. Even when the pKa of the ionizable species in the membrane are known, predicting the ionization state is not straightforward since the pH close to the membrane differs from that in the bulk solution. This is due to the previously described ionic gradient induced by the surface potential: charged species in the membrane attract protons, leading to an acid environment that in turn affects the ionization state of the species at the membrane, generating a feedback loop. Therefore, the fraction of ionized molecules in the membrane is lower than that expected considering bulk pH [21,22,23]. Very interesting, this has the consequence of broadening the shape of the curve of degree of ionization as a function of pH. Furthermore, as the ion cloud thickness depends on ionic strength, the difference between local pH and bulk pH also depends on the salt concentration in the solution [21].

It was shown in the pioneering work by Smith and La Celle [51] that the deformability of erythrocytes changed markedly between pH 5 and 6. In the case of cells, this deformability is potentially related not only to changes in the mechanical properties of the lipid bilayer but also to other changes such as in the cytoskeleton-membrane interactions. In the case of artificial membranes composed only by lipids, the effect of pH on the viscoelastic properties of the bilayer can be studied separately from the other factors.

The lipids that are candidates to act as pH sensors are phospatidic acid, phosphoinositides, phosphoserines and gangliosides. However, not only lipids with ionizable polar headgroups are affected by pH. Zhou et al. reported reduction in the elasticity of membranes composed of phosphatidylcholine at acid pHs, along with an increment in the generalized polarization of the probe Laurdan [52].

The group of Angelova [53] has deeply studied the effect of pH changes on giant unilamellar vesicles (GUV). They demonstrated that a pH change induces deformations of the whole vesicle, as well as vesicle migration and polarization in membranes with phase separated lipid domains. They showed that pH gradients can induce local dynamical membrane deformations, and they have mimicked the formation and behavior of mitochondrial cristae using purely lipidic biomimetic membranes. A model to describe this phenomenon has been developed. Interesting, local pH-induced tubulation is regulated by the lipid composition of the membrane. A very complete review of the experimental results and the related model has been published by the group [53], and thus, we recommend its reading instead of going deeper in this topic here. Related to mitochondrial membranes, we also recommend the review about the architecture and functions of mitochondrial cristae by F. Joubert and N. Puff that appear in this special issue.

## 4. Effects of Ions Different from Protons

### 4.1. Metal Cations

The association of cations to membrane surfaces occurs both, on anionic and zwitterionic lipid bilayers. The extent of binding is greater for charged interfaces due to the increased cation concentration in the ion cloud near the lipid surface, according to the Gouy-Chapman theory.

There is a general trend that cations bind stronger to ordered than to disordered lipid phase states in anionic membranes. This is due to the smaller mean molecular areas in the ordered phases, leading to larger surface charge densities and consequently more negative surface potentials. However, the relationship between ion affinity and molecular area is intricate, because ionizable lipid density (and the consequent charge density and surface potential) in turn modulates lipid ionization fraction [23]. Furthermore, the dependence of the intrinsic affinity of cations to the surface charge density is non-linear, evidencing that besides the unspecific electrostatic interactions, cation-bilayer binding is also mediated by specific interactions [54,55].

The influence of ion binding on the mechanical properties of membranes is mainly due to changes in lipid packing. These changes result from two opposing contributions. On one hand, cation binding may increase the fraction of ionized polar head groups due to changes in the pKa value as stated in Section 3, leading to an expansion in the lipid molecular area, which stabilizes fluid phases. On the other hand, cations preferentially bind to lipid ordered phases, as already mentioned. This is due to two factors: an unspecific electrostatic association to membranes with higher charge density, that leads to lipid molecular area shrinkage due to membrane charge screening (only for anionic membranes), and a specific polydentate coordination to some functional group of the lipid polar moiety, which may be present in both, charged and zwitterionic membranes. This last interaction involves displacement of hydration water, being in fact, determinant for lipid packing effect. It is expected to be dependent on the specific nature of the ion. The balance between these contributions is generally dominated by the membrane compaction effect, originating shifts in the ordered-disordered phase transition towards higher temperatures [23]. Furthermore, ion binging can trigger fluid-to-gel, gel-to-crystal and even lamellar-to-nonlamellar isothermal phase transitions, depending on the particular membrane state and environmental conditions [56].

Evaluation of hydration levels of lipid polar head groups by polarized infrared spectroscopy indicated that the interaction of Li${}^{+}$, and divalent cations Be${}^{2+}$, Mg${}^{2+}$, Ca${}^{2+}$, Sr${}^{2+}$, Ba${}^{2+}$, Zn${}^{2+}$ and Cu${}^{2+}$ stabilizes the gel phase, and consequently increases the melting temperature. Mg${}^{2+}$, Ca${}^{2+}$, Sr${}^{2+}$ and Ba${}^{2+}$ render lipid carbonyl groups more accessible to water because they interact mainly at the phosphate groups level in the gel phase, dehydrating it. Conversely, Be${}^{2+}$, Zn${}^{2+}$ and Cu${}^{2+}$ dehydrate carbonyls, as well as phosphate groups [55].

The mechanical stability of lipid membranes in the presence of different ions was assessed by atomic force microscopy (AFM) in the force spectroscopy mode, testing the response of phospholipid bilayers under compression. An increase in the mechanical stability with the affinity of the adsorbed ions and ionic strength was observed, due to lipid compaction caused by the screening of the interlipid electrostatic repulsion. In the case of compact bilayers, the Van der Waals interactions between lipid acyl chains are maximized increasing cohesion of the membrane. In the case of zwitterionic lipids, anions of the electrolyte solution also participate in the constitution of electrostatic attractive interactions. The effect is higher in the gel phase than in the liquid crystalline phase, and it is sensitive to the ion size and charge. Membrane stability increases with the size in both series of alkali and alkali earth (Li${}^{+}$< Na${}^{+}$< K${}^{+}$; Mg${}^{2+}$< Ca${}^{2+}$) for the gel phase. However there is no significant effect for Cs${}^{+}$ and Sr${}^{2+}$ on either phase state, nor for the alkali earth in the liquid crystalline phase [57].

Membrane rigidity upon bending, that can be quantified by the mean bending rigidity modulus κ, is partially determined by the surface charge density. This regulates the attenuation of the undulations caused by the repulsion between the charged lipids, in addition to the energy cost of bending the diffuse ionic layer [58,59]. Cation binding has a direct impact on surface charge density of anionic lipid bilayers, and consequently, on the mechanic properties of the membrane. The effect of cation binding to anionic dioleoylphosphatidylglycerol bilayer on κ was assessed by molecularly realistic self-consistent field predictions [60]. κ influences the persistence length at the interface and this, in turn, has a significant impact on the equilibrium radius of vesicles *R*, obtained by several freeze-thaw cycles. Claessen et al. proposed two different regimes due to opposing effects that are in delicate equilibrium upon cation binding to the bilayer, leading to a dual behavior in the bending modulus and vesicle size. In a regime of low ionic concentration, both κ and *R* decrease with the ionic strength, due to a decrease of the Debye length, since the deformation of the double layer bears most of the energy cost of bending the membrane. In the presence of high salt concentration, specific contacts of the hydrated ions dominate, driving an increase in lipid packing due to interlipid charge repulsion screening. Together with ion dehydrating effect on the bilayer, this leads to thicker and stiffer membranes with increasing ionic strength. These result in an increase in κ and in *R*, as well as a shift to higher gel-liquid crystalline phase transition temperature.

Alkali cations, specially Na${}^{+}$ and K${}^{+}$, are the most abundant electrolytes in biological systems. The interaction of these ubiquitously distributed monovalent ions with polar head groups of phospholipid aggregates shows relevant effects in lateral organization, including phase segregation in palmitoyloleoylphosphatidylcholine membranes in the presence of NaCl [61]. A multi-approach study by Böckmann et al. has evidenced by fluorescence correlation spectroscopy and molecular dynamics simulations that lateral self-diffusion of lipids decreases significantly with increasing NaCl concentration. This is due to complexation of sodium ions by tight interaction with lipid carbonyl groups with a mean coordination number of three [62]. This chelation-like complexation stabilizes ordered lipid phases. The reduced lipid mobility is also due to the deep penetration of the ions to the C=O level instead of a superficial adsorption at the phosphate layer [62]. This was further supported by the shift of the C=O infrared absorption band and the lack of influence on the PO${}_{2}^{-}$ antisymmetric stretching bands upon sodium chloride addition [55]. It was postulated that this kind of deep coordination can take place in the case of H${}_{3}$O${}^{+}$ ions as well, explaining previous observation of proton long residence times [63,64]. The interaction between Na${}^{+}$ ions and phosphatidylcholine membranes leads to membrane structural changes such as a 5 % increase in membrane thickness. The total electrostatic profile across the bilayer is not significantly altered [62], in line with the lack of changes in the fluorescence emission of voltage sensitive dyes [65]. However, particular contributions to the potential are substantially perturbed. In this regard, changes in the orientation of the lipid polar head groups lead to an increase in dipolar potential, but this is compensated by the polarization of the first hydration layer and the ion cloud distribution [62].

A comparative study of the configuration and dynamics of alkali Na${}^{+}$ and K${}^{+}$ cations at anionic membrane interfacial region was performed combining single-ion level AFM with molecular dynamics simulations [66]. Formation of spatially correlated nano-domains in dipalmitoylphosphatidic acid bilayers due to interactions at the lipid-electrolyte region was evidenced. While sodium ions adsorb strongly to lipid carbonyl oxygens, in an unstructured thick layer, potassium organizes into two discrete coordination layers, separated by a bridging water layer. The slow kinetics of ionic networks evolution inside the nanodomains due to electrostatic interactions and the hydration water molecules that compete for the polar head groups, locally reduce the effective stiffness of the membrane. This constitutes a spontaneous mechanism to tune interfacial mechanical properties in the nanometric scale. The interaction of Na${}^{+}$ ions with anionic phosphatidylglycerol groups is similar to the interaction with zwitterionic phosphatidylcholine, forming a complex with the carbonyl groups of three lipid molecules. In fact, the formation of clusters of ion-bridged lipids through C=O groups has shown to overcome charge repulsion and leads to counterintuitive smaller average areas for anionic lipid compared to neutral analogs [67].

Divalent ions such as Ca${}^{2+}$, Mg${}^{2+}$ and Zn${}^{2+}$ are biologically relevant as well, not only due to its role as cofactors, but also because of the effect they exert when interacting with the membrane as fusion catalysts, and also as ion bridges. Among divalent cations, Ca${}^{2+}$ has a special relevance in part due to its key role for cell signaling and membrane trafficking pathways. Both these activities are related to its mechanism of interaction with negatively charged lipids, where it induces negative spontaneous local curvature. This was evidenced by the formation of invaginations on GUVs composed of phosphatidylserine and phosphatidylinositol-bisphosphate, upon Ca${}^{2+}$ asymmetry across the membrane at sub-millimolar concentration, likely due to Ca${}^{2+}$-induced lipid clustering [68]. Calcium coordination to lipid carbonyl oxygens occurs sequentially to a preferred number of coordination of four, leading to a great reduction in lipids self-diffusion and a decreased rotational diffusion coefficient, as revealed by molecular dynamics simulations [69]. Membrane remodelling was also attained with Mg${}^{2+}$ and Na${}^{+}$ ions, but with millimolar and tens of millimolar transmembrane gradients requirements respectively, due to the higher energetic cost for ion dehydration. Na${}^{+}$ was postulated to induce positive curvature, opposite to Ca${}^{2+}$ [68]. The weak effects promoted by Mg${}^{2+}$ were also attributed to its small size, that is not enough to perturb lipid intermolecular distance as Ca${}^{2+}$ [57].

The interaction of Ca${}^{2+}$ with anionic lipids such as phosphatidic acid and its derivative diacylglycerol pyrophosphate, which are in turn involved in signaling pathways themselves, is characterized by lipid clustering and membrane stiffening, as demonstrated using Langmuir films [70]. Ca${}^{2+}$ binding interacts in an 1:1 stoichiometry with singly-charged di-basic phosphatidic acid, decreasing the second acidic pKa and forming a neutral lipid:Ca(II) complex [71]. There is not such clear picture about the interaction of diacylglycerol pyrophosphate with Ca${}^{2+}$. However, the overall charge neutralizing effect compacts the film packing giving raise to stiffer membranes for pure diacylglycerol pyrophosphate monolayers, as well as for mixtures of diacylglycerol pyrophosphate and phosphatidic acid. These effects are pH-dependent, being more marked at basic pHs, where deprotonated lipids form expanded structures.

Zn${}^{2+}$ has numerous roles as cofactor and structural stabilizer of diverse proteins. The interaction of Zn${}^{2+}$ with model membranes was also sampled in monolayers of phosphatidic acid and diacylglycerol pyrophosphate [72]. Similar to Ca${}^{2+}$, Zn${}^{2+}$ induced a shrinkage due to the screening of the interlipid charge repulsion, consequently stiffening the monolayers. Furthermore, Zn${}^{2+}$ induces a phase transition in phosphatidic acid films from a liquid-expanded to a liquid-condensed state, not observable in the absence of the ion. The effect is sensitive to pH, being more marked at acidic pHs.

Since the strong condensation effect driven by binding of divalent cations on lipid film surfaces may induce phase transition to ordered phase states, these ions can be considered potent regulators of membrane structure. The regulatory mechanism is finely tuned by cation affinity to membranes. The interplay of lipid molecular area and membrane charge density, that leads to ion adsorption/desorption due to membrane phase and structure changes, originates local gradients of ions concentrations [23,56].

### 4.2. Cationic Peptides

**Figure 2:**
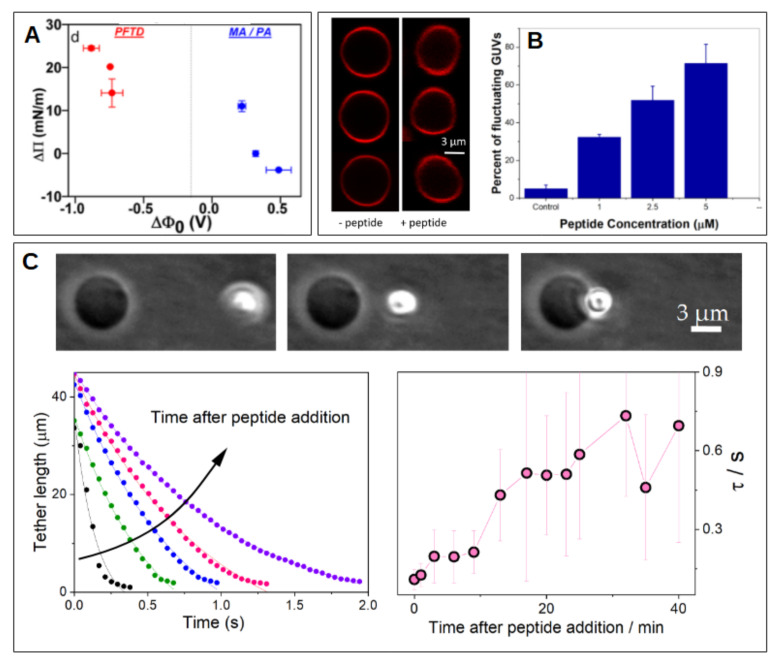
Peptide-membrane interaction. (**A**) Interaction of cationic peptides with lipid monolayers composed of fatty acids. The incorporation (measured as an increase in lateral pressure Δπ) depends on the monolayer dipole potential ($\Delta \Phi_{0}$), and is maximal for systems with inverted (negative) values. Measurements were performed using perfluorotetradecanoic acid (PFTD), myristic acid (MA) or palmitic acid (PA). The plot is adapted with permission from [78]. Copyright (2018) American Chemical Society. (**B**) Shape fluctuations of GUVs in the presence of increasing concentration of a cationic peptide. Adapted from [82]. (**C**) Kinetic of membrane nanotube retraction formed from a GUV by means of optical tweezers. The effect of peptide addition is shown at different times. Adapted from [84] with permision from Elsevier. Copyright Elsevier (2021).

Other very important cation-membrane interaction that is worth mentioning in this review is that between membranes and cationic peptides. Peptides rich in basic aminoacids have shown a high affinity for membranes, which depends on anionic lipids content [73,74,75,76,77,78,79,80], membrane dipole potential [78,81] (see Figure 2A), membrane phase state [82] and chemical composition [78,83,84].

Many of such peptides remodel membrane structure facilitating self- and cargo-permeation, a property that confers them the name of cell-penetrating peptides (CPP). These peptides allow the introduction of small molecules inside cells [85]. The translocation mechanism of CPPs involves membrane softening, leading to membrane protrusion, pore formation or membrane leakage [85,86].

Another family of cationic peptides is that of the antimicrobial peptides (AMPs). They are part of many living organisms immune response, and their antibiotic activity relies on the drastic impact their association has on the membrane structure and integrity [87]. Although treated differently by people interested in CPPs or AMPs, the ability to reach the inner leaflet of lipid bilayers is crucial to both of them. In fact, potentially, all CPPs are AMPs and all AMPs are CPPs, and “membrane-active peptides” (MAPs) would be a better and more inclusive name for all of them [88].

The peptide-induced interfacial remodelling is a combination of electrostatic and topological effects, and this grants many MAPs selectivity to tumor cell membranes rich in glycosaminoglycans [89], and towards microbial over mammalian membranes [74].

There is a considerable amount of evidence of membrane softening, thinning, and changes in fluidity and local curvature by peptide inclusion. Figure 2B,C show examples of membrane softening determined in GUVs for a CPP (B) and an AMP (C). Membrane disorder induced by the peptide has been proposed as the cause of softening [90,91], as well as a decrease in the lipid bilayer cohesion [92]. Membrane thinning, and in turn softening, has been proposed to be due to hydrophobic matching between peptide and membrane [93]. Surface entropy of membrane-bound peptides has also been considered [94]. Peptides that are adsorbed at, or inserted in the membrane, may form clusters [95], and induce structural rearrangements of the lipids around them [96]. This last proposal explains both, membrane softening and stiffening (which is also observed despite not so frequently). Agrawal et al. proposed that each protein has a unique mechanical signature dictated by its specific interfacial coupling to the surrounding membrane [96]. The lipid properties are also crucial because the same peptide may induce softening or stiffening depending on lipid composition, and even depending on peptide-to-lipid ratio [84].

Membrane stiffening due to peptide binding can also be explained considering curvature effects in the membrane due to MAP-lipid structures [97,98,99,100]. Zemel et al. used a molecular-level chain packing theory [101], and found that the curvature stress depends on the penetration depths of the peptide. Interesting, despite thinning of the membrane upon peptide insertion, they found an increase in the bending stiffness.

In addition to the electrostatic-induced adsorption of MAPs to anionic membranes, specific peptide-membrane interactions have been reported, which depend on the aminoacid: polylysine and polyarginine behave differently. Although negative curvature is induced when the polypeptide inserts in the membrane, polyarginine induces simultanueously a positive curvature along one principal direction resulting in a negative Gaussian curvature and a saddle-shapped deformation. This was attributed to the bridging of multiple lipid headgroups by bidentate complexation of arginine with phospate groups [102].

Decoupling of the two leaflets of the bilayer due to peptide insertion has also been considered to account for membrane softening [103]. Furthermore, the formation of hydrophilic pores was considered by Grasso et al., who found, using all atom molecular dynamics simulations that water penetration promoted by MAPs leads to a local decrease of the lipid order, which emerges macroscopically as a reduction of the membrane bending modulus [104].

Lipid recruiting due to a selective interaction of the peptide with a particular lipid class has also been considered [82,105]. Related to this, a theoretical model was proposed that considers electrostatic interactions between MAPs and anionic lipids, leading to membrane disruption [106]. MAPs are proposed to adsorb electrostatically at the membrane interface, and subsequently insert at the lipid head group-tail interface, screening charged lipid-lipid electrostatic repulsion, and inducing an average shrinkage in their molecular area, thus reducing the average area of one of the hemilayers, and originating curvature.

Despite membrane softening due to the interaction with cationic peptides is well reported, and it is accepted that electrostatic interactions play a fundamental role in the interaction, not much discussion about a direct correlation between electrostatics and membrane mechanical properties is found in literature, being the work of Cahill an interesting exception [107]. In this article, the transient pores induced by CPPs are compared to electroporation (see Section 5.2), and a model of transduction is proposed in which phosphatidylserines and CPPs form two plates of a capacitor with a voltage sufficient to form pores due to electroporation [107].

## 5. Potentials across Membranes

### 5.1. Electromechanical Coupling

Potential-induced membrane deformation due to ionic gradients across the bilayer has been experimentally evidenced since late 1960s, when birefringence changes in the axon membrane of neurons was attributed to changes in the potential difference [108]. The electrical response of membranes to a mechanical deformation gives rise to piezoelectricity and flexoelectricity. While piezoelectricity refers to polarization due to uniform strain, flexoelectricity refers specifically to polarization due to strain that changes from point to point in the membrane. In quasi two-dimensional systems such as bio-membranes, piezoelectricity is described as the area-dependence of polarization, while flexoelectricity is the curvature-dependence of the electric field. They are somehow interrelated as membrane curvature can arise from asymmetric changes in the area in one hemilayer relative to the other.

Both flexoelectricty and piezoelectricity manifest bidirectionally: a mechanic strain induces an electric field (direct), and conversely, the electric field induces deformations (converse). These phenomena are involved in membrane electromotility, whose magnitude and polarity depends on membrane stiffness and surface potential [109,110]. This is the origin of complex cellular functions such as mechanosensitivity and mechanotransduction of signals in the nervous system [110]. It is also responsible for electromotility that leads to mammalian hearing [110,111]. Besides understanding its physiological implications, flexoelectricity studies in biomembranes have a further impact on the development of engineering applications based on chemical biosensing [112,113,114,115].

**Figure 3:**
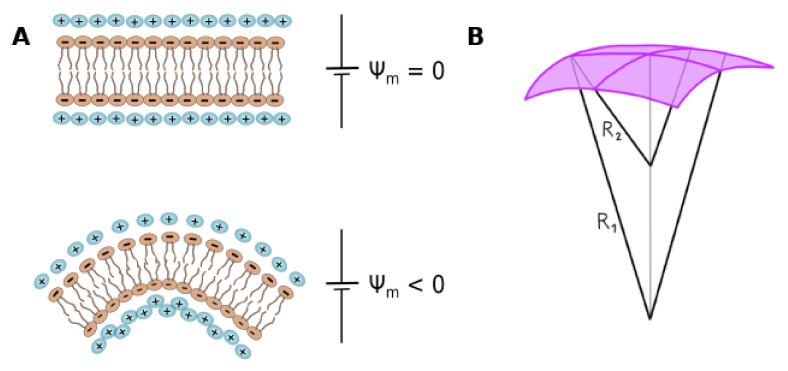
Flexoelectricity. (**A**) Scheme of flexoelectricity: Membrane is plane in the absence of a membrane electric field ($\Psi_{m}=0$), while a curvature is induced due to the effect of the non-zero potential. (**B**) Scheme of curvature definition in a bidimensional plane, where $R_{1}$ and $R_{2}$ are the two main curvature radii.

Flexoelectricity arises from an inhomogeneous electric force across the membrane due to the interaction between the electric field and the molecular multipoles forming the membrane, as well as the ions bound to these molecules. This force induces curvature changes that can be described by Equation (2) [110,112], as represented in Figure 3A:

(2) $$c_{1}+c_{2}=\frac{f}{\kappa }E_{m}$$

where κ is the bending rigidity, $c_{1}$ and $c_{2}$ are the two principal membrane curvatures, with $c_{1}=1/R_{1}$ and $c_{2}=1/R_{2}$ (being $R_{1}$ and $R_{2}$ the two principal radii of curvature schematized in Figure 3B); *f* is the flexoelectric coefficient, defined positive if the polarization points outwards the membrane curvature. *f* is mainly determined by $\Psi_{s}$ and $\Psi_{d}$ [110,112]. $E_{m}$ is the transmembrane electric field, which can be applied externally or can be a consequence of the local distribution of charges. As pointed out previously, spontaneous curvature arises if there is charge asymmetry between membrane surfaces, due to either unequal charged lipid composition in each hemilayer, or ion gradient generated between both ionic diffuse layers. This gives rise to an imbalance in surface pressure (tension) between the two interfaces, producing an emergent curvature [109,116]. In the case of uniformly charged symmetric membranes, instability with respect to a spherical deformation has been proposed for highly charged membranes [117]. This explains the process of spontaneous vesiculation of ionizable membranes upon pH changes. Related to this, changes in the pH (and local dielectric constant) close to the polar headgroups have been reported to depend on the curvature for inverse micelles, and also on the internal leaflet (negative curvature) of anionic liposomes [118]. Interesting, no variation in the local pH with curvature was detected in the external leaflet of the liposomes or in micelles (non-inverted). Therefore, the nature of interfacial curvature geometry and its magnitude contributes to the pH-deviation from the bulk phase to the interface [118].

The first experimental approach to study flexoelectricity in membranes was attained by AC currents registration on planar lipid bilayers subjected to a hydrostatic oscillating strain [119,120]. These experiments were refined and a detailed explanation was provided by Petrov’s group [121,122]. This group also made determinations of flexoelectricity in cell membranes applying the patch-clamp technique [123]. Membrane electromotility due to flexoelectric effect has been determined using AFM and voltage-clamped cells. Voltage induced cantilever movement due to an imbalance in surface tension in the two hemilayers of the bilayer was modelled by Lippman equation [109,111]:

(3) $$\frac{d\gamma }{d\Psi_{s}}=-\Gamma_{q}$$

where γ is the surface tension and $\Gamma_{q}$ is the excess of mobile charges in the proximity of the membrane surface. Relying on the capacitor model to approximate membrane behavior, Lippman equation integration yields:

(4) $$\gamma =-\frac{1}{2}C_{D}\Psi_{s}^{2}+\gamma_{0}$$

$\gamma_{0}$ is the voltage independent tension and $C_{D}$ is the specific capacitance of the ionic layer. This capacitance can be obtained by the Hemholtz’s parallel plate model considering the Debye length as the interplate separation: $C_{D}=\varepsilon_{w}\varepsilon_{0}/\lambda_{D}$ (with $\varepsilon_{w}$ water dielectric constant). This yields $C_{D}\sim$ 30 F cm${}^{-2}$ at physiological saline concentration [110]. The membrane is conceptualized as three serial capacitors: a Debye capacitance for the interfacial ion cloud at each side of the membrane, and a hydrocarbon capacitor in between, with a low capacitance of $C_{m}\sim$ 0.5 F cm${}^{-2}$. Even when neglecting ion adsorption to the membrane, hydration layers, and the discreetness of the charges (and thereby steric hindrance), it has proven to perform nicely on modelling electrical behavior in membranes and electromechanical coupling [124].

Main theoretical approaches to study flexoelectricity in membranes based on continuum models were inspired by the previous observation. Ambjörnsson et. al have derived a continuum model to describe the electromechanical effect of the application of a static potential across the membrane [125]. To this end, the Poisson-Boltzmann equation was resolved in the Debye-Hückel regime for a simplified geometry: a flat non-conductive incompressible membrane between two flat electrodes with applied potential. Curvature changes were allowed by introducing a perturbation of first order term in the electrostatic potential. The membrane mechanical parameters such as the bending rigidity and surface tension were derived from the calculation of the restoring forces in the linear response approximation, valid in the regime of low amplitude undulation. These calculations yielded a positive contribution of the electric field to the bending rigidity, and negative contribution to the membrane tension, implying that an applied potential makes the membrane stiffer towards bending and with lower surface tension. The magnitude of the effect is dependent on the Debye screening layer thickness. This arises from the balance of the free energy contributions due to the compression and expansion of the charge densities at both sides of the bilayer, with opposite sign and different magnitude, and gives rise to the cost aforementioned of bending the ion cloud along with the membrane.

Despite the success of the continuum approach derived by Ambjörnsson et al., Harland et al. reported flaws in performance in continuum models, thus evidencing that electromechanical coupling can be commanded by voltage-dependent discreet adsorption of ions to the phospholipid polar head groups [126]. Neglecting the discreteness of charges leads to lower values for the flexoelectric coefficients compared to experimental values [123]. Even when the obtained *f* resulted smaller than the experimental ones, the flexoelectric effect evidenced by continuum models remained qualitatively satisfactory. Dynamic coupled effects such as viscoelasticity, neglected as well in continuum models, could further contribute to the discrepancies observed.

### 5.2. External Electric Fields and Electroporation

External applied voltages induce mechanical modifications in membranes that can be predicted understanding membrane flexoelectricity and piezoelectricity properties. These mechanical changes in the lipid bilayer are in turn responsible for protein conformational modulation of ion channels and transporters [127].

The effects promoted by external potentials are not different from those related to intrinsic membrane potentials arising from the dipolar nature of the amphiphiles, membrane asymmetries and ion imbalance across the bilayer [128]. There is however a consideration that has to be made in relation to the orientation of the external potential relative to membrane orientation. While membrane potentials keep isotropic symmetry across the membrane all along the interface, external potential adds up depending on the relative local orientation of the membrane [129]. For spheroidal membranes, a uniform externally applied field would vary in intensity and even in sign along the membrane. Bending rigidity is experimentally determined, among other techniques, using the electrodeformation due to converse flexoelectricity. This method relies on the fact that the only possible steady shape of a nearly-spherical GUV in a uniform applied electric field is a prolate ellipsoid. The prolate vesicle deformation results from nonuniform radial electric pressure which modifies the mechanical equilibrium condition for a quasi-spherical vesicle [116,130].

The model derived by Ambjörnsson et al. previously mentioned was proposed for membrane asymmetric charge compositions, and the external potential induces an imbalance in charge densities at both sides of the membrane, enhancing the difference in the free energy contributions by compression-expansion process due to interfacial undulation. At sufficiently large potentials, this energy imbalance occurs even in the symmetric membranes. The decrease in surface tension could exceed the threshold value that the membrane can attain, leading to leakage through pore formation [125]. This electrostatic field-induced transient increase in permeability of cell membranes is known as electroporation and is largely used to introduce small molecules into the cell interior [131,132,133,134,135,136,137]. Loading of cells in suspension by applying voltage pulses involves large electric fields in the aqueous electrolyte (of the order $10^{5}$ V/m for mammalian cells of 10 m). Since a uniform applied potential affects whole cells with variable intensity and directionality, the effect of a pulse in cells depends on the cell geometry. Bacteria are about an order of magnitude smaller than mammalian cells, and require a field around ten fold larger in order to induce similar increases in membrane permeability [134].

Cell membranes are much more complex than a lipid bilayer structure. However, when developing a model it is helpful to start from a simplified one, and the effect of protein inclusions in membrane electroporation is usually excluded, as the lipid bilayer appears to be responsible in electroporation phenomena. The most accepted model considers the existence of transient aqueous pores, with a probability of existence that depends on thermal energy and on the cost of pore formation [138]. Therefore, membrane mechanical properties play an important role in electroporation: deformability of membrane can affect the direction of water penetration [139] and lateral compressibility affects the free energy of pore formation since when the pore is created, its environment is compressed [140].

External potential increases the probability of formation of hydrophilic pores due to a decrease in the energy required for their formation, which is a consequence of changes in the membrane capacitance as pores are filled with water of higher dielectric constant than lipid, among other factors [129,138,141]. Therefore, in the presence of the electric field, poration rate increases and thereby, membrane permeability.

Aside from capacitance changes due to the hydrophilic pores, the field may interact with the molecules that compose the membrane, since they are charged or with an asymmetrical charge density distribution. Besides, the gradient in the dielectric constant within the membrane leads to electro-mechanical Maxwell stresses [6]. Taking these factors into account, it has been proposed that the field induces a decrease in the membrane surface tension to zero, resulting in the total loss of cohesion of the lipid molecules [142]. Additionally, molecular dynamics simulations indicate that the external field can cause rearrangements of ions, hydration water and polar headgroups, leading to more frequent water protrusions into the hydrophobic core of the membrane as compared to the unperturbed case. Water molecules eventually span the whole bilayer and a pore is formed [143]. Electrically induced chemical changes of membrane lipids or proteins may also contribute to the increase in bilayer’s permeability [136]. In this regard, it has been demonstrated that the electric field indirectly facilitates lipid oxidation in liposomes [144].

It was shown that the threshold potential for electroporation of a membrane depends not only on its capacitance and dipole potential but also on the nature of lipids’ hydrophobic tails and polar headgroups, an interesting summary of the findings can be found in ref. [136]. Cholesterol is known to decrease membrane permeability, and consequently increase the threshold potential for electroporation [136,145]. Other sterols and also hopanoids decrease membrane permeability [146], but their effect on electroporation has not been systematically studied. Regarding polar headgroup, increases in the proportion of negatively charged lipids in a membrane increases the rate of pore formation [147]. In relation to phase state, it has been observed that pores form in the disordered phase in membranes with phase separation [148], and even when forcibly created in the ordered phase, they migrate to the disordered one [135].

### 5.3. Nerve Impulse Propagation

Mechanical changes coupled to the changes in membrane potential have been reported over many years using a wide variety of techniques and cell types. Among studies in the topic it is worth citing Tasaki and Byrne [149] that used nonmyelinated nerve fibers. The authors introduced a bundle of three olfactory nerves of garfish into a water-tight chamber, and measured rapid changes in the hydrostatic pressure with a mechanoelectric transducer. They observed that nerves undergo rapid volume expansion while carrying an impulse, due to lateral expansion of the excited portion of the fibers, where the superficial layer is transformed into a low-density structure. Zhang et al. used AFM to record dynamic membrane displacements in voltage-clamped human embryonic kidney cells [109]. Their results indicate that the direction of the displacement depends on ionic strength, and that the amplitude is proportional to voltage.

The group of Anvari developed an experimental setup that combines optical tweezers with patch-clamp. Using optically trapped beads, they detached the plasma membrane from the cytoskeleton and formed membrane tethers, and determined forces at piconewton scale while changing membrane potential [150]. Using this approach, they measured electromechanical force generation by membranes of outer hair cells and human embryonic kidney cells [151]. They found that the mechanical properties of the membrane that determine the force required to pull and maintain membrane tether are a function of the transmembrane electric field.

A different approach was implemented by Akkin et al. These authors used spectral domain optical coherence tomography in order to obtain real-time cross-sectional images of nerves [152]. The system yields subnanometer axial resolution and submillisecond temporal resolution for the measurement. They acquired transient signals from squid giant axons during the propagation of action potentials at room and cold temperatures and in normal and hypertonic solutions, and found alterations in the magnitude and shape of these signals upon changes in temperature and NaCl levels [153].

Oh et al. used a microscope that combines optical interferometry with microscopy, and measured the retardation of the optical phase of the light that traverses a monolayer of human embryonic kidney cells. They observed cell deformation in response changes in the membrane potential caused by isovolumetric cell deformation through a direct coupling between membrane potential and membrane tension [154].

Nguyen et al. measured mechanical deformations of P12 cell lines in response to electrical excitations using piezoelectric nanoribbons. They found cell deflections of 1 nm when 120 mV was applied to the cell membrane. Their results can be explained considering that depolarization caused by the applied voltage induces changes in membrane tension that generate an alteration in cell radius so that the pressure remains constant across the membrane [155].

Yang et al. quantified membrane displacement in cultures of hippocampal neurons using bright field optical image and a differential detection algorithm [156]. They found a maximum amplitude of membrane displacement of ~0.2 nm at ~90 mV depolarization potential, that took place at the peak of the action potential. Very interesting, their method also provides valuable local information. Their experiments revealed large variability in the local membrane displacement, not only in the amplitude but also in the polarity. Local displacement along the edge of a neuron was shown to be negative in some regions, indicating shrinking of the neuron. In other regions, however, the displacement was positive, as expected in the case of expansion or swelling of the neuron. The same research group also proposed a plasmonic imaging method to image mechanical deformation in single cells upon a change in the membrane potential [157].

Full-field imaging of primary neuronal cultures isolated from rat cortical tissues during an action potential was performed using high-speed quantitative phase imaging by Ling. et al. [158]. This technique enables 0.1 ms temporal resolution and an axial path length sensitivity of 4 pm per pixel. Finally, mechanical changes have also been investigated during action potential propagation in plant cells. Plants do not have nerves, but their cells are capable of generating action potentials, just as nerve cells in animals do. Fillafer et al. used micropipette aspiration experiments on Chara braunii internodes [159].

**Figure 4:**
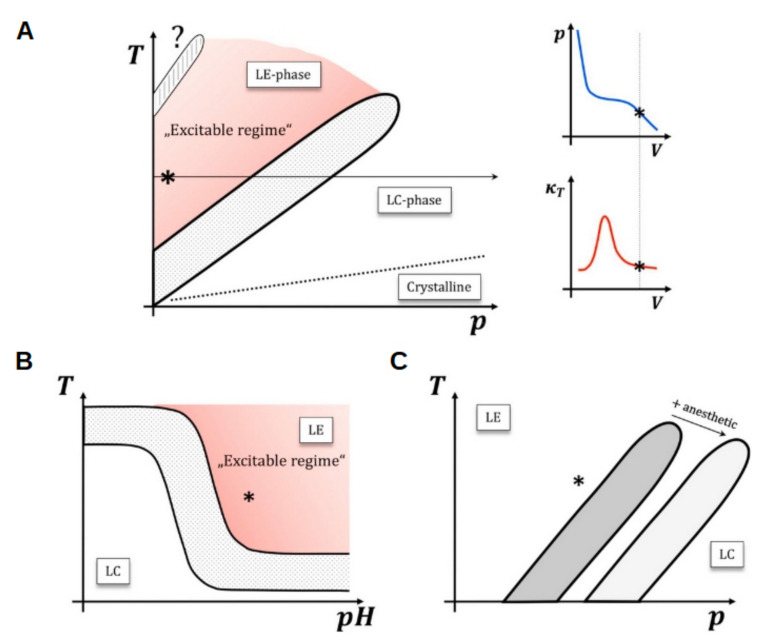
Scheme showing the relation between membrane phase state and excitability. (**A**) The cell membrane is excitable if the resting state (asterisk) is in the vicinity of an ordered-disordered transition (e.g., from liquid expanded (LE) to liquid condensed phase (LC) in liquid monolayers; or from lamellar fluid to gel in lipid bilayers). This is illustrated by the P-V isotherm and the derived isothermal compressibility on the right (the arrow illustrates the respective slice through the phase plane). State changes move the system state (asterisk) through phase space and hence change the physical properties of the membrane. At low T / high P the membrane “freezes” into a crystalline-like state. (**B**) In a T-pH plot, the phase boundary is sigmoidal. The underlying reason for this additional nonlinearity is that the headgroups of membrane molecules are ionizable. This results in a nonlinear change of the transition temperature with pH. Thus, acidification at constant T and P can move the resting state into the LC phase. (**C**) According to the melting point depression theory [162,166], anesthetics leave the resting state in the disordered phase, but increase its distance to the transition. This figure is reproduced from ref. [164] with permission from Elsevier.

Not only electromechanical signals have been reported but also changes in the fluorescence of labelled amphiphiles and in turbidity [160]. Furthermore, it was reported that there is no net heat release after completion of an action potential [161], which is a response that lacks a satisfactory explanation within Hodgkin–Huxley model. Considering these observations, a thermodynamic model of nerve propagation that incorporates phase transitions due to electrostatic changes at the membrane was proposed by Heimburg [24,162,163]. This model is based on the nonlinear electro-mechanical properties caused by the melting transition of biological membranes from an ordered and compact to a disordered fluid state. Ordered membranes display a larger volume and area density than disordered, and therefore, phase transition can be induced by surface pressure changes. Close to transitions, the response of the membrane to changes in lateral pressure is nonlinear, giving rise to the emergence of nonlinear sound pulses that propagate in the membrane. Heimburg and Jackson related these pulses to solitons or solitary waves [162]. The features of these pulses depend largely on the properties of the melting transition in the membranes and, in particular, on the distance of the transition from physiological conditions. Within this frame, cell membranes are excitable if the resting state is in the vicinity of an ordered-disordered transition. Any variable that influences the position of the transition relative to physiological conditions potentially affects the excitability of the membrane, as shown in Figure 4A,B, and clearly explained by Fillafer et al. [164]. In line with this, the Meyer-Overton correlation, known for a long time, states that the potency of general anesthetics is proportional to their solubility in the lipid membrane [165]. Since these molecules dissolve mainly in the fluid state, they affect the thermodynamics of phase transition as shown in Figure 4C, thus inducing a melting-point depression and providing an explanation for the action of this kind of anesthetics [166].

Mechanical vibrations (ultrasound) can modulate action potential in neurons in vitro and in vivo. This coupling has recently been demonstrated in neurons at the single cell level using an interesting setup [167]. When an acoustic wave interacts with the cellular membrane, the ultrasound energy is transferred from one molecule of the material to the next, resulting in a pressure wave. Ultrasonic radiation force causes oscillation and displacement of protein-free model membranes, resulting in changes in membrane area and capacitance [168]. The ultrasound-induced membrane perturbation might interfere with the mechanical waves of spontaneous action potentials, either facilitating or inhibiting them. As such, ultrasound neurostimulation holds great potential as a safe non-invasive brain stimulation approach for basic human neuro-science research and as a clinical intervention tool for psychiatric and neurological disorders [169].

While Heimburg and Jackson´s model focuses on lipids, Hodgkin–Huxley model focuses on proteins, and integrating unifying models have been proposed [170,171]. However, it is certainly important to keep in mind that models are simplified representations of a system, and they are used to represent a phenomenon for a particular purpose, but they do not necessarily describe accurately and completely the real system. An interesting discussion related to this can be found in ref. [172].

All in all, it is beyond doubt that during the action potential, both the thickness and the area of the cell membrane change in comparison to the resting membrane, generating a density pulse. Furthermore, nonlinear pulses with the same characteristics as action potentials have been excited in phospholipid monolayers devoid of protein at the air-water interface [173]. Thus, electromechanical response of lipidic membranes is important and explains, at least partially, electric signal propagation in neurons, providing a useful model in neuroscience.

Related to the changes in membrane potential during the propagation of the electric pulse, the Hodgkin–Huxley model makes use of the Goldman–Hodgkin–Katz equation, which assumes that at rest, the net current flowing through the membrane is zero, and predicts that the membrane potential is commanded by the permeability of the membrane to each ion, which in turn governs ion fluxes. In contrasts to this interpretation, Tamagawa et al. proposed an alternative explanation, considering that ions adsorb at the membrane surface (without crossing it) [174]. As already stated in Section 4, ion affinity to the membrane depends on the particular ion and on the membrane composition. Besides, it varies with phase state, and according to Tamagawa et al., this would lead to changes in the membrane potential.

## 6. Summary and Future Perspectives

A large amount of experimental and theoretical data has been gathered up to now related to the behavior of both, lipid bilayers and cell membranes, under external and internal electric fields. These fields affect membrane stiffness, curvature and permeability. Electroporation has been widely studied, as well as pH and cation binding effects on membrane deformations, and density pulses due to electric fields. However, there are scarce reports aiming to include all effects together, and the currently available information is scattered throughout reports in different research fields of membrane biophysics.

We summarize here some observations performed on cell membranes and on lipid bilayers, with the aim of clustering the information. We found that in general the correlation between membrane electrostatics and mechanical properties upon some environmental changes is accurately modelled by fundamental theories, such as electromechanical coupling due to potentials across the membrane using Lippman equation or flexoelectricity formalism. However, there are some other background factors, such as the effect of charged species adsorption, that are scarcely correlated in a systematic manner. This is not due to a capricious bias in the literature, but has deep roots in the discreteness of charged chemical species. When assessing macroscopic effects, this discreteness of the charged species translates into singularities in membrane mechanical response upon the interaction with each species. It would be very important to span the greatest amount of chemical species in systematic studies, and to establish robust patterns.

The take-home message of this review is that, when working with cell membranes, it is necessary to bear in mind that the effects mentioned in each section of this work may occur simultaneously, and locally distributed in different regions of the membrane. Each effect is a simple physicochemical phenomenon in essence, and constitutes the fine-tuning spontaneous mechanism of the cell to regulate very complex phenomena such as electromotility and nerve impulse propagation. The orchestrated spacial and temporal interconnection of all the simultaneous effects breeds the cellular membrane complexity. This makes the understanding of processes very intricate when assessing living cells, but at the same time extremely interesting and challenging.

## Data Availability

Not applicable.

## References

[1] Israelachvili J.N. (2011). Intermolecular and Surface Forces.

[2] Montich G.G., Bustos M.M., Maggio B., Cumar F.A. (1985). Micropolarity of interfaces containing anionic and neutral glycosphingolipids as sensed by Merocyanine 540. Chem. Phys. Lipids.

[3] Cooke R., Kuntz I. (1974). The properties of water in biological systems. Annu. Rev. Biophys. Bioeng..

[4] Mild K.H., Løvtrup S. (1985). Movement and structure of water in animal cells. Ideas and experiments. Biochim. Et Biophys. Acta Rev. Biomembr..

[5] Bagatolli L.A., Stock R.P., Olsen L.F. (2019). Coupled response of membrane hydration with oscillating metabolism in live cells: An alternative way to modulate structural aspects of biological membranes?. Biomolecules.

[6] Timoshkin I., MacGregor S., Fouracre R., Given M., Anderson J. Forces acting on biological cells in external electrical fields. Proceedings of the 2006 IEEE Conference on Electrical Insulation and Dielectric Phenomena.

[7] Elia S., Lamberti P., Tucci V. A finite element model for the axon of nervous cells. Proceedings of the COMSOL Conference.

[8] Singh S., Krishnaswamy J.A., Melnik R. (2020). Biological cells and coupled electro-mechanical effects: The role of organelles, microtubules, and nonlocal contributions. J. Mech. Behav. Biomed. Mater..

[9] Honrado C., Ciuffreda L., Spencer D., Ranford-Cartwright L., Morgan H. (2018). Dielectric characterization of Plasmodium falciparum-infected red blood cells using microfluidic impedance cytometry. J. R. Soc. Interface.

[10] Gascoyne P.R., Noshari J., Becker F.F., Pethig R. (1994). Use of dielectrophoretic collection spectra for characterizing differences between normal and cancerous cells. IEEE Trans. Ind. Appl..

[11] Asami K., Hanai T., Koizumi N. (1976). Dielectric properties of yeast cells. J. Membr. Biol..

[12] Raicu V., Raicu G., Turcu G. (1996). Dielectric properties of yeast cells as simulated by the two-shell model. Biochim. Et Biophys. Acta Bioenerg..

[13] Haandbæk N., Bürgel S.C., Heer F., Hierlemann A. (2014). Characterization of subcellular morphology of single yeast cells using high frequency microfluidic impedance cytometer. Lab A Chip.

[14] Gagnon Z., Gordon J., Sengupta S., Chang H.C. (2008). Bovine red blood cell starvation age discrimination through a glutaraldehyde-amplified dielectrophoretic approach with buffer selection and membrane cross-linking. Electrophoresis.

[15] Huang Y., Wang X.B., Gascoyne P.R., Becker F.F. (1999). Membrane dielectric responses of human T-lymphocytes following mitogenic stimulation. Biochim. Et Biophys. Acta Biomembr..

[16] Hughes M.P., Morgan H., Rixon F.J., Burt J.P., Pethig R. (1998). Manipulation of herpes simplex virus type 1 by dielectrophoresis. Biochim. Et Biophys. Acta Gen. Subj..

[17] Huang Y., Wang X.B., Holzel R., Becker F., Gascoyne P. (1995). Electrorotational studies of the cytoplasmic dielectric properties of Friend murine erythroleukaemia cells. Phys. Med. Biol..

[18] Kleijin J., Van Leeuwen H. (2000). Electrostatic and Electrodynamic Properties of Biological Interphases.

[19] Clarke R.J. (2001). The dipole potential of phospholipid membranes and methods for its detection. Adv. Colloid Interface Sci..

[20] Yi M., Nymeyer H., Zhou H.X. (2008). Test of the Gouy-Chapman theory for a charged lipid membrane against explicit-solvent molecular dynamics simulations. Phys. Rev. Lett..

[21] Wilke N. (2015). Monomolecular Films of Surfactants with Phase-Coexistence: Distribution of the Phases and Their Consequences. Comprehensive Guide for Nanocoatings Technology. Vol 2: Characterization and Reliability.

[22] Mercado F.V., Maggio B., Wilke N. (2011). Phase diagram of mixed monolayers of stearic acid and dimyristoylphosphatidylcholine. Effect of the acid ionization. Chem. Phys. Lipids.

[23] Träuble H. (1977). Membrane electrostatics. Structure of Biological Membranes.

[24] Heimburg T. (2008). Thermal Biophysics of Membranes.

[25] Eisenberg M., Gresalfi T., Riccio T., McLaughlin S. (1979). Adsorption of monovalent cations to bilayer membranes containing negative phospholipids. Biochemistry.

[26] Egorova E., Dukhin A., Svetlova I. (1992). Some problems of zeta potential determination in electrophoretic measurements on lipid membranes. Biochim. Et Biophys. Acta Biomembr..

[27] McDaniel R.V., McLaughlin A., Winiski A.P., Eisenberg M., McLaughlin S. (1984). Bilayer membranes containing the ganglioside GM1: Models for electrostatic potentials adjacent to biological membranes. Biochemistry.

[28] Simon S., McIntosh T. (1989). Magnitude of the solvation pressure depends on dipole potential. Proc. Natl. Acad. Sci. USA.

[29] Li A.J., Nussinov R. (1998). A set of van der Waals and coulombic radii of protein atoms for molecular and solvent-accessible surface calculation, packing evaluation, and docking. Proteins Struct. Funct. Bioinform..

[30] Parsons D.F., Ninham B.W. (2009). Ab initio molar volumes and Gaussian radii. J. Phys. Chem. A.

[31] Woodle M., Collins L., Sponsler E., Kossovsky N., Papahadjopoulos D., Martin F. (1992). Sterically stabilized liposomes. Reduction in electrophoretic mobility but not electrostatic surface potential. Biophys. J..

[32] Freire J.M., Domingues M.M., Matos J., Melo M.N., Veiga A.S., Santos N.C., Castanho M.A. (2011). Using zeta-potential measurements to quantify peptide partition to lipid membranes. Eur. Biophys. J..

[33] Tocanne J.F., Teissié J. (1990). Ionization of phospholipids and phospholipid-supported interfacial lateral diffusion of protons in membrane model systems. Biochim. Et Biophys. Acta Rev. Biomembr..

[34] Tsui F.C., Ojcius D.M., Hubbell W.L. (1986). The intrinsic pKa values for phosphatidylserine and phosphatidylethanolamine in phosphatidylcholine host bilayers. Biophys. J..

[35] Egorova E.M. (1996). Determination of the apparent pK values of ionizable groups in highly charged lipid membranes. Colloids Surfaces A Physicochem. Eng. Asp..

[36] Egorova E.M. (1998). Dissociation constants of lipid ionizable groups I. Corrected values for two anionic lipids. Colloids Surfaces A Physicochem. Eng. Asp..

[37] Egorova E. (1998). Dissociation constants of lipid ionizable groups II. Changes in surface pK at low ionic strengths. Colloids Surfaces A Physicochem. Eng. Asp..

[38] Kanicky J.R., Shah D.O. (2002). Effect of degree, type, and position of unsaturation on the pKa of long-chain fatty acids. J. Colloid Interface Sci..

[39] Kanicky J., Poniatowski A., Mehta N., Shah D. (2000). Cooperativity among molecules at interfaces in relation to various technological processes: Effect of chain length on the p K a of fatty acid salt solutions. Langmuir.

[40] Pashkovskaya A.A., Vazdar M., Zimmermann L., Jovanovic O., Pohl P., Pohl E.E. (2018). Mechanism of long-chain free fatty acid protonation at the membrane-water interface. Biophys. J..

[41] Kanicky J., Shah D. (2003). Effect of premicellar aggregation on the p K a of fatty acid soap solutions. Langmuir.

[42] Le Calvez E., Blaudez D., Buffeteau T., Desbat B. (2001). Effect of cations on the dissociation of arachidic acid monolayers on water studied by polarization-modulated infrared reflection-absorption spectroscopy. Langmuir.

[43] Lakhdar-Ghazal F., Tichadou J.L., Tocanne J.F. (1983). Effect of pH and Monovalent Cations on the Ionization State of Phosphatidylglycerol in Monolayers: An Experimental (Surface Potential) and Theoretical (Gouy-Chapman) Approach. Eur. J. Biochem..

[44] Van Dijck P., De Kruijff B., Verkleij A., Van Deenen L., De Gier J. (1978). Comparative studies on the effects of pH and Ca^2+^ on bilayers of various negatively charged phospholipids and their mixtures with phosphatidylcholine. Biochim. Et Biophys. Acta.

[45] Mengistu D.H., Kooijman E.E., May S. (2011). Ionization properties of mixed lipid membranes: A Gouy–Chapman model of the electrostatic–hydrogen bond switch. Biochim. Et Biophys. Acta Biomembr..

[46] Loew S., Kooijman E.E., May S. (2013). Increased pH-sensitivity of protein binding to lipid membranes through the electrostatic-hydrogen bond switch. Chem. Phys. Lipids.

[47] Graber Z.T., Thomas J., Johnson E., Gericke A., Kooijman E.E. (2018). Effect of H-bond donor lipids on phosphatidylinositol-3, 4, 5-trisphosphate ionization and clustering. Biophys. J..

[48] Gilbile D., Docto D., Kingi D., Kurniawan J., Monahan D., Tang A., Kuhl T. (2019). How Well Can You Tailor the Charge of Lipid Vesicles?. Langmuir.

[49] Pace C.N., Grimsley G.R., Scholtz J.M. (2009). Protein ionizable groups: PK values and their contribution to protein stability and solubility. J. Biol. Chem..

[50] Pahari S., Sun L., Alexov E. (2019). PKAD: A database of experimentally measured pKa values of ionizable groups in proteins. Database.

[51] Smith B.D., la Celle P.L. (1979). Parallel decrease of erythrocyte membrane deformability and spectrin solubility at low pH. Blood.

[52] Zhou Y., Raphael R.M. (2007). Solution pH alters mechanical and electrical properties of phosphatidylcholine membranes: Relation between interfacial electrostatics, intramembrane potential, and bending elasticity. Biophys. J..

[53] Angelova M.I., Bitbol A.F., Seigneuret M., Staneva G., Kodama A., Sakuma Y., Kawakatsu T., Imai M., Puff N. (2018). pH sensing by lipids in membranes: The fundamentals of pH-driven migration, polarization and deformations of lipid bilayer assemblies. Biochim. Et Biophys. Acta Biomembr..

[54] Mclaughlin S., Mulrine N., Gresalfi T., Vaio G., Mclaughlin A. (1981). Adsorption of divalent cations to bilayer membranes containing phosphatidylserine. J. Gen. Physiol..

[55] Binder H., Zschörnig O. (2002). The effect of metal cations on the phase behavior and hydration characteristics of phospholipid membranes. Chem. Phys. Lipids.

[56] Cevc G. (1991). Isothermal lipid phase transitions. Chem. Phys. Lipids.

[57] Redondo-Morata L., Oncins G., Sanz F. (2012). Force spectroscopy reveals the effect of different ions in the nanomechanical behavior of phospholipid model membranes: The case of potassium cation. Biophys. J..

[58] Vitkova V., Cenova J., Finogenova O., Mitov M., Ermakov Y., Bivas I. (2004). Surface charge effect on the bending elasticity of lipid bilayers. C. R. L’Academie Bulg. Sci..

[59] Winterhalter M., Helfrich W. (1992). Bending elasticity of electrically charged bilayers: Coupled monolayers, neutral surfaces, and balancing stresses. J. Phys. Chem..

[60] Claessens M., Leermakers F., Hoekstra F., Cohen Stuart M. (2007). Opposing effects of cation binding and hydration on the bending rigidity of anionic lipid bilayers. J. Phys. Chem. B.

[61] Rappolt M., Pabst G., Amenitsch H., Laggner P. (2001). Salt-induced phase separation in the liquid crystalline phase of phosphatidylcholines. Colloids Surfaces A Physicochem. Eng. Asp..

[62] Böckmann R.A., Hac A., Heimburg T., Grubmüller H. (2003). Effect of sodium chloride on a lipid bilayer. Biophys. J..

[63] Alexiev U., Mollaaghababa R., Scherrer P., Khorana H., Heyn M. (1995). Rapid long-range proton diffusion along the surface of the purple membrane and delayed proton transfer into the bulk. Proc. Natl. Acad. Sci. USA.

[64] Heberle J., Riesle J., Thiedemann G., Oesterhelt D., Dencher N.A. (1994). Proton migration along the membrane surface and retarded surface to bulk transfer. Nature.

[65] Clarke R.J., Lüpfert C. (1999). Influence of anions and cations on the dipole potential of phosphatidylcholine vesicles: A basis for the Hofmeister effect. Biophys. J..

[66] Trewby W., Faraudo J., Voïtchovsky K. (2019). Long-lived ionic nano-domains can modulate the stiffness of soft interfaces. Nanoscale.

[67] Zhao W., Róg T., Gurtovenko A.A., Vattulainen I., Karttunen M. (2007). Atomic-scale structure and electrostatics of anionic palmitoyloleoylphosphatidylglycerol lipid bilayers with Na^+^ counterions. Biophys. J..

[68] Graber Z., Shi Z., Baumgart T. (2017). Cations induce shape remodeling of negatively charged phospholipid membranes. Phys. Chem. Chem. Phys..

[69] Böckmann R.A., Grubmüller H. (2004). Multistep binding of divalent cations to phospholipid bilayers: A molecular dynamics study. Angew. Chem.-Int. Ed..

[70] Margutti M.P., Wilke N., Villasuso A.L. (2020). Influence of Ca^2+^ on the surface behavior of phosphatidic acid and its mixture with diacylglycerol pyrophosphate at different pHs. Chem. Phys. Lipids.

[71] Laroche G., Dufourc E.J., Dufourcq J., Pezolet M. (1991). Structure and dynamics of dimyristoylphosphatidic acid/calcium complexes by deuterium NMR, infrared, and Raman spectroscopies and small-angle X-ray diffraction. Biochemistry.

[72] Villasuso A.L., Wilke N., Maggio B., Machado E. (2014). Zn^2+^-dependent surface behavior of diacylglycerol pyrophosphate and its mixtures with phosphatidic acid at different pHs. Front. Plant Sci..

[73] Ben-Tal N., Honig B., Peitzsch R.M., Denisov G., McLaughlin S. (1996). Binding of small basic peptides to membranes containing acidic lipids: Theoretical models and experimental results. Biophys. J..

[74] Paterson D.J., Tassieri M., Reboud J., Wilson R., Cooper J.M. (2017). Lipid topology and electrostatic interactions underpin lytic activity of linear cationic antimicrobial peptides in membranes. Proc. Natl. Acad. Sci. USA.

[75] Derakhshankhah H., Jafari S. (2018). Cell penetrating peptides: A concise review with emphasis on biomedical applications. Biomed. Pharmacother..

[76] Ruseska I., Zimmer A. (2020). Internalization mechanisms of cell-penetrating peptides. Beilstein J. Nanotechnol..

[77] Alvares D.S., Viegas T.G., Neto J.R. (2017). Lipid-packing perturbation of model membranes by pH-responsive antimicrobial peptides. Biophys. Rev..

[78] Via M.A., del Pópolo M.G., Wilke N. (2018). Negative Dipole Potentials and Carboxylic Polar Head Groups Foster the Insertion of Cell-Penetrating Peptides into Lipid Monolayers. Langmuir.

[79] Via M.A., Klug J., Wilke N., Mayorga L.S., del Pópolo M.G. (2018). The interfacial electrostatic potential modulates the insertion of cell-penetrating peptides into lipid bilayers. Phys. Chem. Chem. Phys..

[80] Via M.A., Wilke N., Mayorga L.S., del Pópolo M.G. (2020). Surface charge density and fatty acids enhance the membrane permeation rate of CPP–cargo complexes. Soft Matter.

[81] Batta G., Karpati L., Fulaneto G., Tarapcsak S., Kovacs T., Zakany F., Mandity I., Nagy P. (2020). Statin-boosted cellular uptake and endosomal escape of penetratin due to reduced membrane dipole potential. Authorea Prepr..

[82] Crosio M.A., Via M.A., Cámara C.I., Mangiarotti A., del Pópolo M.G., Wilke N. (2019). Interaction of a polyarginine peptide with membranes of different mechanical properties. Biomolecules.

[83] Sharmin S., Islam M.Z., Karal M.A.S., Alam Shibly S.U., Dohra H., Yamazaki M. (2016). Effects of lipid composition on the entry of cell-penetrating peptide oligoarginine into single vesicles. Biochemistry.

[84] Alvares D.S., Monti M.R., Neto J.R., Wilke N. (2021). The antimicrobial peptide Polybia-MP1 differentiates membranes with the hopanoid, diplopterol from those with cholesterol. BBA Adv..

[85] Herce H., Garcia A., Litt J., Kane R., Martín P., Enrique N., Rebolledo A., Milesi V. (2009). Arginine-rich peptides destabilize the plasma membrane, consistent with a pore formation translocation mechanism of cell-penetrating peptides. Biophys. J..

[86] Di Pisa M., Chassaing G., Swiecicki J.M. (2015). Translocation mechanism (s) of cell-penetrating peptides: Biophysical studies using artificial membrane bilayers. Biochemistry.

[87] Kristiansen J.E., Hendricks O., Delvin T., Butterworth T.S., Aagaard L., Christensen J.B., Flores V.C., Keyzer H. (2007). Reversal of resistance in microorganisms by help of non-antibiotics. J. Antimicrob. Chemother..

[88] Henriques S.T., Melo M.N., Castanho M.A. (2006). Cell-penetrating peptides and antimicrobial peptides: How different are they?. Biochem. J..

[89] Jobin M.L., Alves I.D. (2014). On the importance of electrostatic interactions between cell penetrating peptides and membranes: A pathway toward tumor cell selectivity?. Biochimie.

[90] Akabori K., Huang K., Treece B.W., Jablin M.S., Maranville B., Woll A., Nagle J.F., Garcia A.E., Tristram-Nagle S. (2014). HIV-1 Tat membrane interactions probed using X-ray and neutron scattering, CD spectroscopy and MD simulations. Biochim. Et Biophys. Acta Biomembr..

[91] Tristram-Nagle S., Chan R., Kooijman E.E., Qiang W., Weliky D.P., Nagle J.F. (2011). HIV Fusion Peptide Penetrates, Disorders and Softens T-Cell Membrane Mimics. Biophys. J..

[92] Fa N., Lins L., Courtoy P.J., Dufrêne Y., van der Smissen P., Brasseur R., Tyteca D., Mingeot-Leclercq M.P. (2007). Decrease of elastic moduli of DOPC bilayers induced by a macrolide antibiotic, azithromycin. Biochim. Et Biophys. Acta Biomembr..

[93] Pan J., Tieleman D.P., Nagle J.F., Kučerka N., Tristram-Nagle S. (2009). Alamethicin in lipid bilayers: Combined use of X-ray scattering and MD simulations. Biochim. Et Biophys. Acta Biomembr..

[94] Pabst G., Danner S., Podgornik R., Katsaras J. (2007). Entropy-driven softening of fluid lipid bilayers by alamethicin. Langmuir.

[95] Häckl W., Seifert U., Sackmann E. (1997). Effects of fully and partially solubilized amphiphiles on bilayer bending stiffness and temperature dependence of the effective tension of giant vesicles. J. Phys. II.

[96] Agrawal H., Zelisko M., Liu L., Sharma P. (2016). Rigid proteins and softening of biological membranes—with application to HIV-induced cell membrane softening. Sci. Rep..

[97] Bouvrais H., Méléard P., Pott T., Jensen K.J., Brask J., Ipsen J.H. (2008). Softening of POPC membranes by magainin. Biophys. Chem..

[98] Fournier J. (1996). Nontopological saddle-splay and curvature instabilities from anisotropic membrane inclusions. Phys. Rev. Lett..

[99] Vitkova V., Méléard P., Pott T., Bivas I. (2006). Alamethicin influence on the membrane bending elasticity. Eur. Biophys. J..

[100] West A., Brummel B.E., Braun A.R., Rhoades E., Sachs J.N. (2016). Membrane remodeling and mechanics: Experiments and simulations of *α*-Synuclein. Biochim. Et Biophys. Acta Biomembr..

[101] Zemel A., Ben-Shaul A., May S. (2008). Modulation of the spontaneous curvature and bending rigidity of lipid membranes by interfacially adsorbed amphipathic peptides. J. Phys. Chem. B.

[102] Mishra A., Gordon V.D., Yang L., Coridan R., Wong G.C. (2008). HIV TAT forms pores in membranes by inducing saddle-splay curvature: Potential role of bidentate hydrogen bonding. Angew. Chem. Int. Ed..

[103] Shchelokovskyy P., Tristram-Nagle S., Dimova R. (2011). Effect of the HIV-1 fusion peptide on the mechanical properties and leaflet coupling of lipid bilayers. New J. Phys..

[104] Grasso G., Muscat S., Rebella M., Morbiducci U., Audenino A., Danani A., Deriu M.A. (2018). Cell penetrating peptide modulation of membrane biomechanics by Molecular dynamics. J. Biomech..

[105] Dupuy F.G., Pagano I., Andenoro K., Peralta M.F., Elhady Y., Heinrich F., Tristram-Nagle S. (2018). Selective interaction of colistin with lipid model membranes. Biophys. J..

[106] Taheri-Araghi S., Ha B.Y. (2010). Cationic antimicrobial peptides: A physical basis for their selective membrane-disrupting activity. Soft Matter.

[107] Cahill K. (2009). Simple model of the transduction of cell-penetrating peptides. IET Syst. Biol..

[108] Cohen L., Keynes R., Hille B. (1968). Light scattering and birefringence changes during nerve activity. Nature.

[109] Zhang P.C., Keleshian A.M., Sachs F. (2001). Voltage-induced membrane movement. Nature.

[110] Sachs F., Brownell W., Petrov A. (2009). Membrane electromechanics in biology, with a focus on hearing. MRS Bull..

[111] Zhang R., Qian F., Rajagopalan L., Pereira F.A., Brownell W.E., Anvari B. (2007). Prestin modulates mechanics and electromechanical force of the plasma membrane. Biophys. J..

[112] Petrov A.G. (1999). The Lyotropic State of Matter: Molecular Physics and Living Matter Physics.

[113] Vaseashta A.K., Mihailescu I.N. (2008). Functionalized Nanoscale Materials, Devices and Systems.

[114] Rey A.D., Servio P., Herrera-Valencia E. (2013). Bioinspired model of mechanical energy harvesting based on flexoelectric membranes. Phys. Rev. E.

[115] Ahmadpoor F., Sharma P. (2015). Flexoelectricity in two-dimensional crystalline and biological membranes. Nanoscale.

[116] Vlahovska P.M. (2015). Voltage-morphology coupling in biomimetic membranes: Dynamics of giant vesicles in applied electric fields. Soft Matter.

[117] May S. (1996). Curvature elasticity and thermodynamic stability of electrically charged membranes. J. Chem. Phys..

[118] Sarkar Y., Majumder R., Das S., Ray A., Parui P.P. (2017). Detection of curvature-radius-dependent interfacial pH/polarity for amphiphilic self-assemblies: Positive versus negative curvature. Langmuir.

[119] Pasechnik V., Sokolov V. (1973). Change in the permeability of modified bimolecular phospholipid membranes iwth periodic stratching. Biofizika.

[120] Ochs A.L., Burton R.M. (1974). Electrical response to vibration of a lipid bilayer membrane. Biophys. J..

[121] Petrov A.G., Vassileva J. (1975). Physical and Chemical Bases of Biological Information Transfer.

[122] Derzhanski A., Petrov A., Pavloff Y. (1981). Curvature induced conductive and displacement currents through lipid bilayers. J. Phys. Lett..

[123] Petrov A.G. (2001). Flexoelectricity of model and living membranes. Biochim. Et Biophys. Acta Biomembr..

[124] Winiski A.P., McLaughlin A.C., McDaniel R.V., Eisenberg M., McLaughlin S. (1986). An experimental test of the discreteness-of-charge effect in positive and negative lipid bilayers. Biochemistry.

[125] Ambjörnsson T., Lomholt M.A., Hansen P.L. (2007). Applying a potential across a biomembrane: Electrostatic contribution to the bending rigidity and membrane instability. Phys. Rev. E.

[126] Harland B., Brownell W.E., Spector A.A., Sun S.X. (2010). Voltage-induced bending and electromechanical coupling in lipid bilayers. Phys. Rev. E.

[127] Bezanilla F. (2008). How membrane proteins sense voltage. Nat. Rev. Mol. Cell Biol..

[128] Pearlstein R.A., Dickson C.J., Hornak V. (2017). Contributions of the membrane dipole potential to the function of voltage-gated cation channels and modulation by small molecule potentiators. Biochim. Et Biophys. Acta Biomembr..

[129] Weaver J.C., Chizmadzhev Y.A. (1996). Theory of electroporation: A review. Bioelectrochem. Bioenerg..

[130] Dimova R., Riske K.A., Aranda S., Bezlyepkina N., Knorr R.L., Lipowsky R. (2007). Giant vesicles in electric fields. Soft Matter.

[131] Zimmermann U., Vienken J., Pilwat G. (1980). Development of drug carrier systems: Electrical field induced effects in cell membranes. J. Electroanal. Chem. Interfacial Electrochem..

[132] Neumann E., Schaefer-Ridder M., Wang Y., Hofschneider P. (1982). Gene transfer into mouse lyoma cells by electroporation in high electric fields. EMBO J..

[133] Needham D., Hochmuth R. (1989). Electro-mechanical permeabilization of lipid vesicles. Role of membrane tension and compressibility. Biophys. J..

[134] Weaver J.C. (2003). Electroporation of biological membranes from multicellular to nano scales. IEEE Trans. Dielectr. Electr. Insul..

[135] Martí J.M.L., English N.J., del Pópolo M.G. (2018). Elucidating mysteries of phase-segregated membranes: Mobile-lipid recruitment facilitates pores’ passage to the fluid phase. Phys. Chem. Chem. Phys..

[136] Kotnik T., Rems L., Tarek M., Miklavčič D. (2019). Membrane electroporation and electropermeabilization: Mechanisms and models. Annu. Rev. Biophys..

[137] Kirsch S.A., Böckmann R.A. (2019). Coupling of membrane nanodomain formation and enhanced electroporation near phase transition. Biophys. J..

[138] Chen C., Smye S., Robinson M., Evans J. (2006). Membrane electroporation theories: A review. Med. Biol. Eng. Comput..

[139] Sun S., Yin G., Lee Y.K., Wong J.T., Zhang T.Y. (2011). Effects of deformability and thermal motion of lipid membrane on electroporation: By molecular dynamics simulations. Biochem. Biophys. Res. Commun..

[140] Hoejholt K., Mužić T., Jensen S., Dalgaard L.T., Bilgin M., Nylandsted J., Heimburg T., Frandsen S., Gehl J. (2019). Calcium electroporation and electrochemotherapy for cancer treatment: Importance of cell membrane composition investigated by lipidomics, calorimetry and in vitro efficacy. Sci. Rep..

[141] Neu W.K., Neu J.C. (2009). Theory of electroporation. Cardiac Bioelectric Therapy.

[142] Lewis T.J. (2003). A model for bilayer membrane electroporation based on resultant electromechanical stress. IEEE Trans. Dielectr. Electr. Insul..

[143] Böckmann R.A., de Groot B.L., Kakorin S., Neumann E., Grubmüller H. (2008). Kinetics, statistics, and energetics of lipid membrane electroporation studied by molecular dynamics simulations. Biophys. J..

[144] Breton M., Mir L.M. (2018). Investigation of the chemical mechanisms involved in the electropulsation of membranes at the molecular level. Bioelectrochemistry.

[145] Van Uitert I., le Gac S., van den Berg A. (2010). Determination of the electroporation onset of bilayer lipid membranes as a novel approach to establish ternary phase diagrams: Example of the l-*α*-PC/SM/cholesterol system. Soft Matter.

[146] Mangiarotti A., Genovese D.M., Naumann C.A., Monti M.R., Wilke N. (2019). Hopanoids, like sterols, modulate dynamics, compaction, phase segregation and permeability of membranes. Biochim. Et Biophys. Acta Biomembr..

[147] Ahamed M.K., Karal M.A.S., Ahmed M., Ahammed S. (2020). Kinetics of irreversible pore formation under constant electrical tension in giant unilamellar vesicles. Eur. Biophys. J..

[148] Sengel J.T., Wallace M.I. (2016). Imaging the dynamics of individual electropores. Proc. Natl. Acad. Sci. USA.

[149] Tasaki I., Byrne P. (1990). Volume expansion of nonmyelinated nerve fibers during impulse conduction. Biophys. J..

[150] Qian F., Ermilov S., Murdock D., Brownell W.E., Anvari B. (2004). Combining optical tweezers and patch clamp for studies of cell membrane electromechanics. Rev. Sci. Instrum..

[151] Brownell W.E., Qian F., Anvari B. (2010). Cell membrane tethers generate mechanical force in response to electrical stimulation. Biophys. J..

[152] Akkin T., Joo C., de Boer J.F. (2007). Depth-resolved measurement of transient structural changes during action potential propagation. Biophys. J..

[153] Akkin T., Landowne D., Sivaprakasam A. (2009). Optical coherence tomography phase measurement of transient changes in squid giant axons during activity. J. Membr. Biol..

[154] Oh S., Fang-Yen C., Choi W., Yaqoob Z., Fu D., Park Y., Dassari R.R., Feld M.S. (2012). Label-free imaging of membrane potential using membrane electromotility. Biophys. J..

[155] Nguyen T.D., Deshmukh N., Nagarah J.M., Kramer T., Purohit P.K., Berry M.J., McAlpine M.C. (2012). Piezoelectric nanoribbons for monitoring cellular deformations. Nat. Nanotechnol..

[156] Yang Y., Liu X.W., Wang H., Yu H., Guan Y., Wang S., Tao N. (2018). Imaging action potential in single mammalian neurons by tracking the accompanying sub-nanometer mechanical motion. ACS Nano.

[157] Yang Y., Liu X., Wang S., Tao N. (2019). Plasmonic imaging of subcellular electromechanical deformation in mammalian cells. J. Biomed. Opt..

[158] Ling T., Boyle K.C., Zuckerman V., Flores T., Ramakrishnan C., Deisseroth K., Palanker D. (2020). High-speed interferometric imaging reveals dynamics of neuronal deformation during the action potential. Proc. Natl. Acad. Sci. USA.

[159] Fillafer C., Mussel M., Muchowski J., Schneider M.F. (2018). Cell surface deformation during an action potential. Biophys. J..

[160] Tasaki I., Carnay L., Sandlin R., Watanabe A. (1969). Fluorescence changes during conduction in nerves stained with acridine orange. Science.

[161] Abbott B., Hill A.V., Howarth J. (1958). The positive and negative heat production associated with a nerve impulse. Proc. R. Soc. Lond. Ser. B-Biol. Sci..

[162] Heimburg T., Jackson A.D. (2005). On soliton propagation in biomembranes and nerves. Proc. Natl. Acad. Sci. USA.

[163] Heimburg T. (2012). The capacitance and electromechanical coupling of lipid membranes close to transitions: The effect of electrostriction. Biophys. J..

[164] Fillafer C., Paeger A., Schneider M.F. (2020). The living state: How cellular excitability is controlled by the thermodynamic state of the membrane. Prog. Biophys. Mol. Biol..

[165] Overton C.E. (1991). Studies of Narcosis.

[166] Wang T., Mužić T., Jackson A.D., Heimburg T. (2018). The free energy of biomembrane and nerve excitation and the role of anesthetics. Biochim. Et Biophys. Acta Biomembr..

[167] Tamayo-Elizalde M., Chen H., Malboubi M., Ye H., Jerusalem A. (2021). Action potential alterations induced by single F11 neuronal cell loading. Prog. Biophys. Mol. Biol..

[168] Prieto M.L., Oralkan Ö., Khuri-Yakub B.T., Maduke M.C. (2013). Dynamic response of model lipid membranes to ultrasonic radiation force. PLoS ONE.

[169] Jerusalem A., Al-Rekabi Z., Chen H., Ercole A., Malboubi M., Tamayo-Elizalde M., Verhagen L., Contera S. (2019). Electrophysiological-mechanical coupling in the neuronal membrane and its role in ultrasound neuromodulation and general anaesthesia. Acta Biomater..

[170] Engelbrecht J., Peets T., Tamm K., Laasmaa M., Vendelin M. (2018). On the complexity of signal propagation in nerve fibres. Proc. Est. Acad. Sci..

[171] Chen H., Garcia-Gonzalez D., Jérusalem A. (2019). Computational model of the mechanoelectrophysiological coupling in axons with application to neuromodulation. Phys. Rev. E.

[172] Holland L., de Regt H.W., Drukarch B. (2019). Thinking about the nerve impulse: The prospects for the development of a comprehensive account of nerve impulse propagation. Front. Cell. Neurosci..

[173] Shrivastava S., Schneider M.F. (2014). Evidence for two-dimensional solitary sound waves in a lipid controlled interface and its implications for biological signalling. J. R. Soc. Interface.

[174] Tamagawa H., Ikeda K. (2018). Another interpretation of the Goldman–Hodgkin–Katz equation based on Ling’s adsorption theory. Eur. Biophys. J..

